# A Single Lineage of Hepatitis E Virus Causes both Outbreaks and Sporadic Hepatitis in Sudan

**DOI:** 10.3390/v8100273

**Published:** 2016-10-06

**Authors:** Adel Hussein Elduma, Mai Mohammed Adam Zein, Marie Karlsson, Isam M.E. Elkhidir, Heléne Norder

**Affiliations:** 1 National Public Health Laboratory, Ministry of Health—Sudan, 11111 Khartoum, Sudan; dumanet@yahoo.com; 2 Public Health Laboratory, Ministry of Health North Kordofan State—Sudan, 51111 Obeid, Sudan; mailabbb@gmail.com; 3 Department of Infectious Diseases, Institute of Biomedicine at Sahlgrenska Academy, University of Gothenburg, 405 30 Gothenburg, Sweden; marie.karlsson.2@gu.se; 4 Department of Microbiology and Parasitology, Faculty of Medicine, University of Khartoum—Sudan, 11111 Khartoum, Sudan; isamelkhidir@gmail.com

**Keywords:** hepatitis E virus, outbreak, endemic, Sudan, sporadic hepatitis E, HEV

## Abstract

Few studies have reported sporadic hepatitis E virus (HEV) infections during non-outbreak periods in Africa. In this study, the prevalence of HEV infection in Sudan was investigated in 432 patients with acute hepatitis from 12 localities in North Kordofan, and from 152 patients involved in smaller outbreaks of hepatitis in the neighbouring Darfur. HEV infection was diagnosed in 147 (25%) patients: 98 from Kordofan and 49 from Darfur. The mortality was 10%; six of the patients who died from the infection were pregnant women. HEV RNA was detected by quantitative real-time polymerase chain reaction (RT-qPCR) in 38 (26%) patients: 22 from Kordofan and 16 from Darfur. Partial open reading frame (ORF) 1 and ORF2 were sequenced from HEV from nine and three patients, respectively. Phylogenetic analysis showed that the Sudanese strains belonged to genotype 1 (HEV1), and confirmed the segregation of African HEV1 strains into one branch divergent from Asian HEV1. It also revealed that the Sudanese strains from this study and from an outbreak in 2004 formed a separate clade with a common ancestor, distinct from strains from the neighbouring Chad and Egypt. This HEV strain has thus spread in a large area of Sudan, where it has caused both sporadic hepatitis E and outbreaks from at least 2004 and onwards. These data demonstrate that hepatitis E is a constant, on-going public health problem in Sudan and that there is a need for hepatitis E surveillance, outbreak preparedness, and general improvements of the sanitation in these remote areas of the country.

## 1. Introduction

The hepatitis E virus (HEV) is the causative agent of acute hepatitis E. The HEV infection is considered to be endemic in most countries and may cause large outbreaks in Africa and Asia [1].

HEV is a non-enveloped, single-stranded, positive-sense RNA virus belonging to the *Hepeviridae* family. There are four genotypes infecting humans: HEV1–HEV4. HEV1 and HEV2 infect only humans, whereas HEV3 and HEV4 can also cause infections in several other mammalian species, such as domestic pigs, wild boars, rabbits, deer, rats, and mongooses [1].

HEV is usually transmitted through the fecal–oral route [2,3], but may also be blood borne [4,5]; person-to-person transmission is rare [6]. In countries where HEV1 and HEV2 are endemic, HEV may cause widespread outbreaks, mainly through contaminated water. Insufficient drinking water and low standards of sanitation are the main causes of these outbreaks, particularly during heavy rainy seasons when the rivers overflow and drinking water may become contaminated with animal and human fecal matter [7,8].

Various clinical manifestations of HEV, from subclinical to fulminant forms, have been observed. The symptoms include jaundice, dark urine, fever, fatigue, loss of appetite, nausea, vomiting, abdominal pain, and joint pain [9]. HEV1 and HEV2 infections are often observed in individuals between the ages of 15 and 40 years of age [10]. These genotypes have caused widespread and isolated outbreaks in several countries in North and East Africa and in the Middle East [11,12,13,14,15,16,17], and have been shown to be responsible for 20%–60% of all hepatitis cases in these regions. During outbreaks, these genotypes may cause severe hepatitis with a fatality rate of over 10% in pregnant women infected during the third trimester [3].

The first reported cases of HEV infection in Sudan occurred in 1992 [18]; since then several larger outbreaks have been observed, particularly in refugee camps in the Darfur region. Furthermore, all of these outbreaks have been shown to be associated with high mortality rates in pregnant women [19,20,21,22]. Large outbreaks have also been reported in camps with Sudanese refugees in Chad and Ethiopia [23,24,25]. Sudanese HEV strains causing outbreaks among displaced individuals in Darfur in 2004 were shown to belong to genotype 1 [26]. However, little is known about the genetic variability of HEV strains from different regions of Sudan.

The aims of this study were to investigate the prevalence of endemic HEV infection in patients with clinical symptoms of hepatitis during a non-outbreak period in the district of North Kordofan in Central Sudan, and the genetic relationships of these HEV strains with those that have caused outbreaks in Sudan, particularly in the Darfur region.

## 2. Materials and Methods

### 2.1. Patients

**Figure 1:**
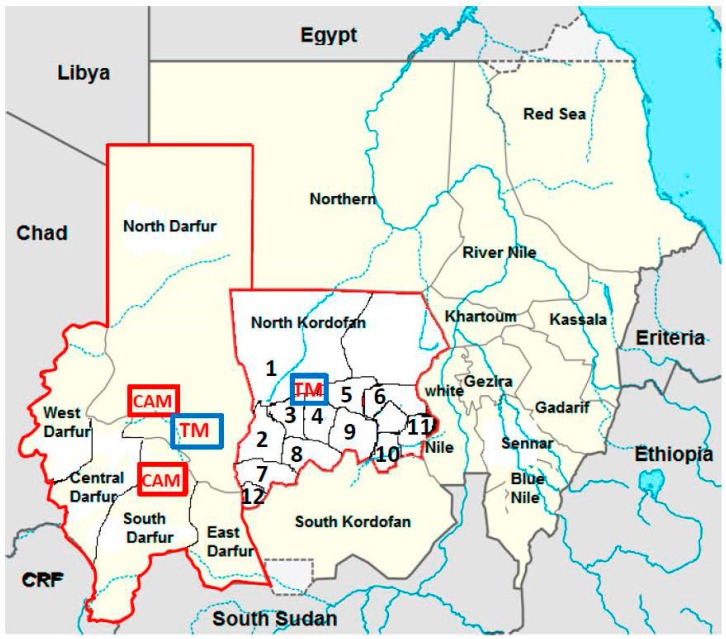
Map of Sudan and the region of North Kordofan and Darfur. The regions for gold mining are shown in squares labelled TM (traditional mining), and the location of the refugee camps in the Darfur regions are shown as squares labelled with CAM (refugee camps). The five regions with hepatitis outbreaks in Darfur are shown in the map as well as the 12 regions of North Kordofan, from where patients with sporadic hepatitis originated. These regions are labelled with the following Arabic numbers on the map: 1—Sudari; 2—Wad Banda; 3—Al Khawai; 4—Alnohod; 5—West Bara; 6—Bara; 7—Gibaish; 8—Abozabad; 9—Shaikan; 10—Alrahad; 11—Omrawaba; and 12—Alodeia.

Because the symptomatic, acute HEV infection is largely indistinguishable from other acute-phase hepatic illnesses, all patients with clinical symptoms of hepatitis were asked to participate in this study during their consultations at health clinics in 12 different localities in the state of North Kordofan in Sudan, between May 2011 and May 2012. Hepatitis symptoms were defined as fever, jaundice, headache, vomiting, loss of appetite, and abdominal pain. The patients were referred to the largest hospital in Kordofan (the Obeid Teaching Hospital, El Obeid). The North Kordofan state covers an area of about 400,000 square kilometres divided into 14 regions (Figure 1), and has a population of 3.6 to 4 million inhabitants, many of whom are nomads. Demographic data and clinical variables were obtained from all patients using a questionnaire designed for this study. National Public Health Laboratory of Sudan has a general approval from the federal ministry of health to conduct any study that can help defining any outbreak further and can help in preventive measures and monitoring outbreaks. All enrolled patients gave their written consent to participate. Patients involved in hepatitis outbreaks between May 2012 and May 2014, in five different states of the Darfur region, were also enrolled. The samples were anonymized with only sex, age, and origin of the patients known. Darfur covers an area of approximately 500,000 square kilometres divided into five regions, and has a population size of about 6 million inhabitants. Since 2003, famine and conflicts have affected the civilians hard in Darfur, where several camps with internally displaced persons are situated.

These studies have been performed according to the World Medical Association Declaration of Helsinki. All serum samples were analyzed at the reference laboratory for hepatitis E diagnostics at the National Public Health Laboratory in Khartoum, Sudan.

### 2.2. Serum Samples

Serum samples from 432 patients from Kordofan and 152 from Darfur were analyzed in this study. Five milliliters of blood were collected from each patient, and the sera were stored at −20 °C at the site of collection until they were sent to the National Public Health Laboratory in Khartoum, where they were stored at −80 °C before analysis.

### 2.3. Analysis for Anti-HEV Antibodies

The MP Diagnostic HEV enzyme-linked immunosorbent assay (ELISA) (MP Biomedical, Santa Ana, CA, USA) was used for anti-HEV immunoglobulin (Ig) M detection in serum samples according to the manufacturer’s instructions. Twenty-nine of the serum samples reactive for anti-HEV IgM were sent for genotyping to the Clinical Microbiology-Virology Laboratory (CMVL) at Sahlgrenska University Hospital, Gothenburg, Sweden. Thirteen of the samples were from sporadic cases from Kordofan and 16 were from patients involved in outbreaks in Darfur. These samples were reanalyzed and confirmed anti-HEV IgG and IgM positive in Sweden with HEV IgM/HEV IgG assays (DiaPro, Milan, Italy).

### 2.4. HEV RNA Detection by Quantitative Real-Time Polymerase Chain Reaction

HEV RNA detection was performed by quantitative real-time polymerase chain reaction (RT-qPCR) at the National Public Health Laboratory in Khartoum. RNA was extracted from 140 μL of serum using a QIAmp Viral RNA Mini Kit (Qiagen GmbH, Hilden, Germany). HEV RNA was detected with the HEV RT-qPCR assay (Genome Diagnostics, New Delhi, India) in a final volume of 25 μL, according to the manufacturer’s instructions, using a Rotor Gene Q 6000 real-time PCR machine for amplification (Corbett Life Science, Düsseldorf, Germany).

### 2.5. PCR Amplification and Sequencing

Extraction of nucleic acids in the 29 anti-HEV IgM positive samples sent to the CMVL in Sweden was performed from a 250 μL serum mixed with 2 mL of lysis buffer (NucliSENSeasyMAG, bioMérieux, Marcy-l’Étoile, France). The mixture was incubated for 10 min at room temperature before the addition of 50 μL of NucliSENSeasyMAG Magnetic Silica and thereafter incubated for an additional 10 min. RNA was eluted in 110 μL of distilled water using the NucliSENSeasyMAG instrument according to the manufacturer’s instructions (bioMérieux).

cDNA synthesis and PCR amplification of the partial open reading frame (ORF) 1 region were performed as previously described [27,28]. The partial ORF2 region (778 nucleotides) was also amplified using 5 μL of cDNA and primers gt1-ORF2-S1: 5’-GCGGCCTACCGACAGAATTGATTTCGTC-3’ (at position 6247 of AY204877) and gt1-ORF2-AS1: 5’-TCCCGAGTTTTACCCACCTTCATYTTAAG-3’ (at position 7053). This product was semi-nested with primers gt1-ORF2-S2: 5’-ACGCCCAGTCGTCTCAGCCAATGG-3’ (at position 6299) and gt1-ORF2-AS1: 5’-TCCCGAGTTTTACCCACCTTCATYTTAAG-3’ (at position 7053) to yield a 778 bp fragment. The amplified fragments were purified and sequenced in both directions with the primers used in the PCR amplification, as previously described [28]. The sequences obtained in this study are deposited in GenBank with accession number KX879758-KX879765.

### 2.6. Phylogenetic Analysis

The sequences obtained were analyzed with the SeqMan program in the DNAStar program package version 10.1.2 (DNA Star Inc, Madison, WI, USA). The sequences were aligned with the corresponding region of 187 sequences representing HEV genotypes 1–4 in ORF1 and 116 sequences representing genotypes 1–4 in ORF2 obtained from GenBank, including all available HEV sequences from Africa. A phylogenetic analysis was carried out with the PHYLIP package version 3.65 [29]. Evolutionary distances were calculated using the Hasegawa–Kishino–Yano (HKY) algorithm in the DNADIST program in the PHYLIP package with a transition/transversion ratio of 7.10 for ORF1 and 4.95 for ORF2 with gamma correction with alpha 0.31 and 0.38 for ORF1 and ORF2, respectively. Phylogenetic trees were constructed using the unweight pair-group method using arithmetic averages (UPGMA) and the neighbor-joining method in the NEIGHBOR program of the PHYLIP package. Bootstrap analyses were performed for 1000 replicas with the program Seqboot in the PHYLIP package. The trees were visualized with the program Tree View, version 1.6.6 [30].

### 2.7. Statistical Analysis

Data were analyzed using the Statistical Package for Social Science (IBM SPSS 20; IBM, Armonk, NY, USA).

## 3. Results

### 3.1. Patients

All patients enrolled in this study, owing to hepatitis-like illnesses, were from 12 regions of North Kordofan and from five states of Darfur (Table 1; Figure 1). The mean age of the 432 patients with sporadic hepatitis was 23.01 ± 14 years (211 men and 221 women), 32 were younger than five years of age (Table 1). The 152 patients involved in hepatitis outbreaks in Darfur were of similar age: mean age 24.8 ± 12 years (123 males and 29 females), with nine children younger than 5 years of age.

### 3.2. Sporadic Cases of HEV Infection in North Kordofan from 2011 to 2012

Anti-HEV IgM, which was used as a marker for ongoing HEV infection, was found in samples from 98 (23%) of the patients with sporadic hepatitis in Kordofan (Table 1), and HEV RNA was identified in 32 of these samples. There were significantly more women than men infected with HEV (63 (28%) versus 35 (17%); *p* = 0.0039, Fisher’s exact test). The prevalence of infected women was highest in Al Khawai (79%), which was significantly higher than in other regions (*p* = 0.0001, Fisher’s exact test; Table 1).

The most common clinical symptoms observed in the 98 patients with sporadic HEV infection were jaundice (97 patients (99%)), followed by fever, abdominal pain, and vomiting (Table 2). All five children younger than 5 years of age with hepatitis E had fever, and all but one also had jaundice and abdominal pain. HEV infection was detected in all age groups (Table 2), there was, however, a significantly higher frequency of HEV infections in patients younger than 35 years of age (*p* = 0.0082; Fisher’s exact test) than in older. About 60% of the infected women were of reproductive age. Twenty-three of the 432 patients with hepatitis died, 10 of whom had hepatitis E, three men, and seven women. These 10 individuals had a median age of 20 years (range 17–35 years) and were from six different regions of North Kordofan. Six of the seven women who did not survive the HEV infection were pregnant.

### 3.3. Outbreaks in Darfur during the Years 2012 and 2014

During the years 2012 and 2014 there were several small outbreaks of hepatitis in each of the five states of Darfur, involving, in total, 152 patients (Table 1). The largest outbreaks occurred in North and South Darfur with 34 and 48 patients with hepatitis, respectively. Anti-HEV IgM could be found in samples from 49 (32%) of the patients involved in these outbreaks. HEV RNA was detected in 18 (37%) of the HEV IgM-positive samples (Table 3). There were more males than females involved in the outbreaks, and subsequently there were more identified HEV infected males than females, 42 versus 7, respectively. Several of these males were working with gold mining (Figure 1). The mean age of all those who were HEV-infected was 26.7 ± 7.5 years; none were younger than 14 years of age.

### 3.4. HEV Genotyping

Partial ORF1 could be amplified in eight of 13 HEV IgM-positive samples from Kordofan and in five of 16 samples from Darfur. This amplified region could be sequenced in nine samples, five from Kordofan and four from Darfur.

**Figure 2:**
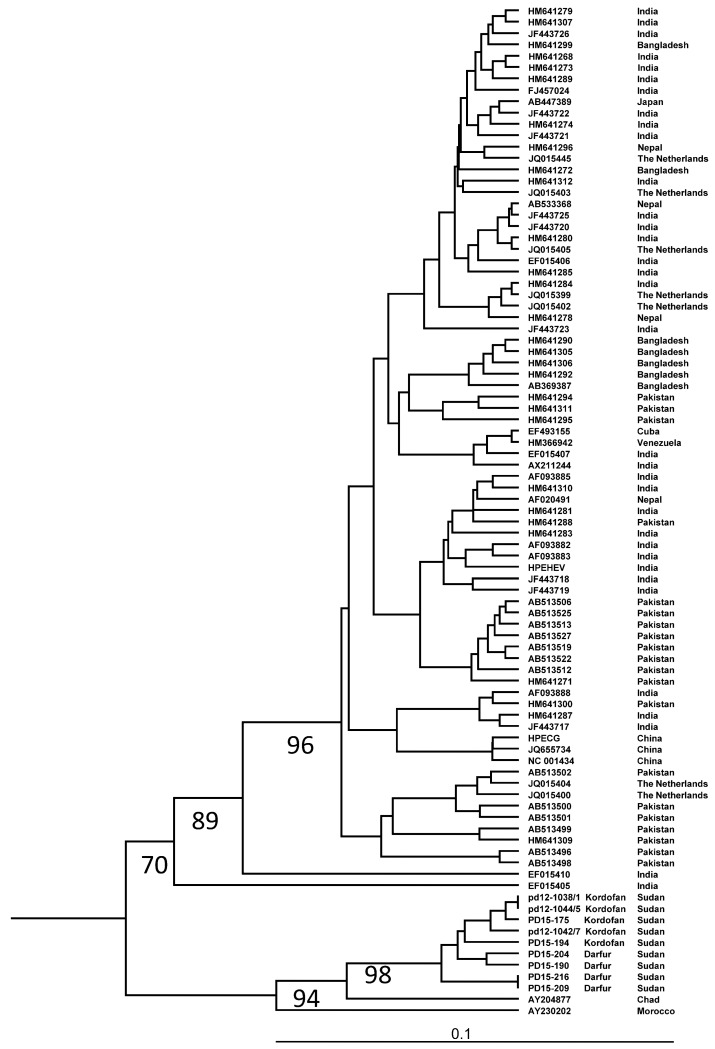
Branch of 89 genotype 1 strains in a phylogenetic tree based on 368 nucleotides of partial polymerases in the open reading frame (ORF) 1 region. Boot strap values obtained from 1000 replicates are given at the branches. The accession numbers and origins of the strains are given at the nodes.

**Figure 3:**
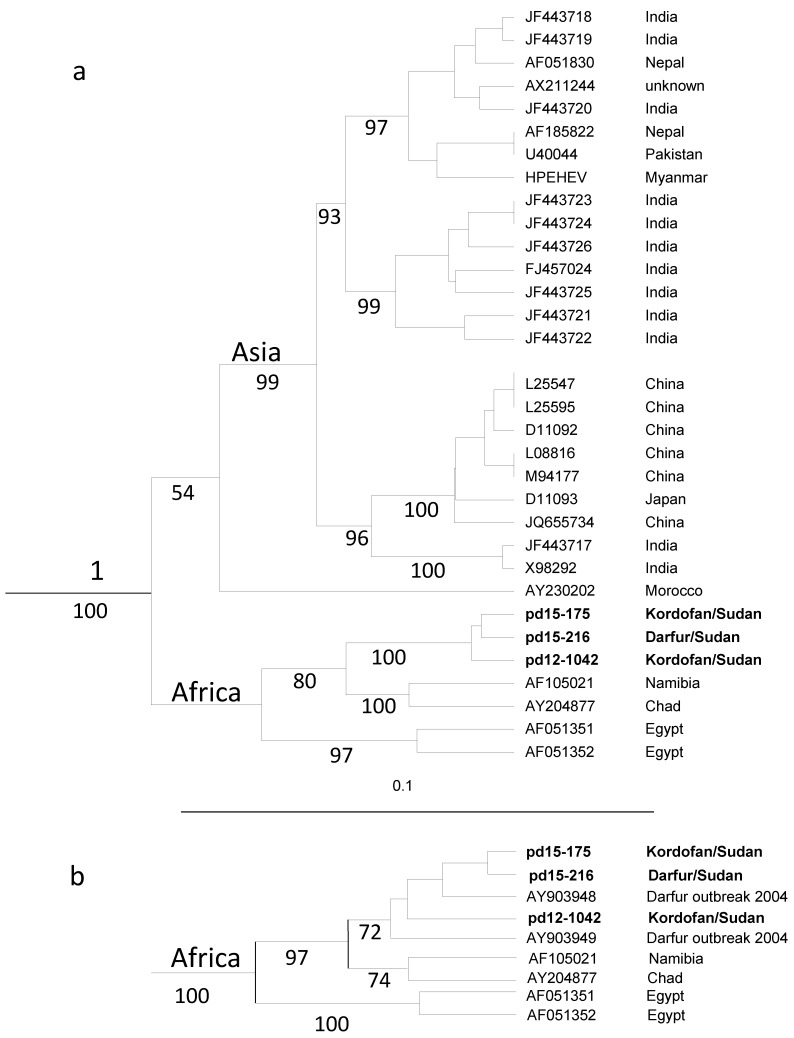
(**a**) Branch formed by 32 HEV genotype 1 (HEV1) strains in a phylogenetic tree based on 658 nucleotides of partial capsids in the ORF2 region; (**b**) Branch formed by the nine available HEV1 sequences from Africa in a phylogenetic tree based on 363 nucleotides of partial capsids in the ORF2 region. Boot strap values obtained from 1000 replicates are given at the branches. The accession numbers and origins of the strains are given at the nodes.

Phylogenetic analysis of partial ORF1 sequences revealed a separate branch within genotype 1 formed by HEV strains from Africa (Figure 2). The strains from Sudan formed a separate clade on this branch, supported by a 98% bootstrap value. There was no separate clade formed by the strains from the outbreaks, and the Sudanese strains were intermixed with each other. An analysis of partial ORF2 sequences confirmed this finding, with the strains from Sudan forming a separate clade on a branch formed by strains from Namibia, Chad and Sudan within genotype 1 supported by a 100% bootstrap value (Figure 3a,b). In this region, the Sudanese strains were 7%–9% divergent from the strains from Namibia, Chad and Egypt, and 13%–15% divergent from the strain from Morocco.

## 4. Discussion

This study showed that hepatitis E is endemic in Sudan, causing sporadic cases during non-outbreak periods as well as larger and smaller outbreaks and is an ongoing public health problem. Sporadic hepatitis E occurred in all localities of North Kordofan where more than seven individuals were investigated, with the highest prevalence in Al Khawai, indicating that there may have been an unidentified outbreak there during the study period. There were also several smaller hepatitis outbreaks in the neighboring Darfur region during the study period, with one third of the cases caused by HEV. The most common symptoms of HEV infection among the sporadic cases were icterus and/or fever and abdominal pain, as has been shown for other clinical hepatitis E cases [31,32], however other symptoms of the disease may have been overlooked, since the patients were selected due to having hepatic symptoms.

Most studies on hepatitis E in Africa have investigated major outbreaks, such as those occurring in Somalia, Botswana, and Namibia in the 1980s, Morocco in 1994, and Central Africa Republic, Uganda, Kenya, and Sudan in the 2000s and 2010s [12,16,21,33,34,35,36,37]. In the current study, we reported the prevalence of HEV infection in regions that have not had any HEV outbreaks, or during periods with no larger outbreaks. One study performed in Northern Uganda after an outbreak in 2009 [38] found ongoing endemic hepatitis E in a large proportion of patients with jaundice, as was found in North Kordofan in this study. In both of these studies, the patients were identified due to symptomatic disease, which has been shown to occur in about 20% of the HEV infected patients in India [10]. If these figures also apply to African countries, the burden of HEV infection is probably substantial, with endemic hepatitis E causing many sporadic cases and unidentified smaller outbreaks, as found in the Kaboong district in Northern Uganda [38] and identified in this study in the Al Khawai region.

There was no difference in age between the genders of the HEV-infected patients. However, there were significantly more women than men with sporadic hepatitis E in Kordofan, while there were more men than women involved in the outbreaks in Darfur. Previous studies have shown that clinical infections are more prevalent in men than in women during outbreaks of HEV [39,40]. The reason for this discrepancy is not known; additional studies including more Sudanese patients are needed in both Northern Kordofan and in neighbouring states during outbreak and non-outbreak periods. It is possible that more women were identified among the sporadic cases because many infected men do not seek health care if the infection is mild and self-limiting, but are tested for HEV during outbreaks. Alternatively, the HEV strain(s) circulating in Sudan may cause more serious infections in women than in men. One reason why most of those involved in the smaller outbreaks in Darfur were men in this study is that the outbreaks occurred in areas where there is a tradition of gold mining, which is mainly performed by men. In these areas the men live in non-hygienic conditions, in crowded places, without latrines and may easily become infected by the fecal–oral route.

Most cases of acute HEV infection were in those between 15 and 24 years old of age, an age also described for sporadic hepatitis E cases in India [10], and about 30% of the children with hepatitis examined in this study were infected with HEV. This mean age is somewhat higher than what has been described from larger outbreaks in Africa, where the average age of infection was 8.1 years in North Africa and 15.5 years in Sub-Saharan Africa [41]. This may be because children can more easily become infected during outbreaks, which often occur in densely populated areas with crowded living conditions that may facilitate multiple transmission routes. Moreover, families are more likely to seek out healthcare during an outbreak than in an endemic situation with sporadic transmission of HEV.

In this study, the mortality rate among Sudanese patients with sporadic hepatitis E was high: 5.3%; 43% of them were due to an HEV infection. Among the HEV infected the mortality was 10%, which is higher than the 0.4%–4% reported for several outbreaks [41,42]. However, during the outbreak in Darfur in 2004, the mortality rate was even higher (17.8%) [21]. Additionally, 60% of the individuals who did not survive their HEV infection in this study were pregnant women, which may explain the high mortality rate, since HEV1 infections in Africa and Asia have been shown to be associated with a 10-fold higher risk of death in pregnant women than in non-pregnant individuals [41]. However, we could not determine how many of the HEV-infected women were pregnant, which makes the interpretation uncertain regarding mortality of pregnant HEV infected women in this study.

The high prevalence of endemic cases of HEV infection in North Kordofan, close to the Darfur region, may indicate that endemic HEV strains can spread and cause outbreaks, such as those occurring in resource-limited regions or in refugee camps, where there is overcrowding and limited access to potable water, proper sanitation, and hygiene. At least three widespread outbreaks have occurred under these conditions in Darfur in recent years [21,26,43,44], and during this study period there were several smaller hepatitis outbreaks in this region. The majority of the inhabitants in North Kordofan are poor and live in conditions similar to those of a study in Uganda [45]. Most are nomads, moving their animals from one region to another. Their water sources are scarce; some have wells, others store rain water for drinking. Additionally, very few individuals have access to latrines. In rural areas, there are no pipe water networks, and only some families have access to traditional latrines. Most fecal–oral spread outbreaks start during the rainy season, often during heavy flooding, which is associated with the spread of HEV and other infectious agents, including cholera, as has been described in Uganda [46,47]. Thus, these conditions may explain the spread of infections from one region to another and may allow for outbreaks to occur if the herd immunity is low in the population.

A phylogenetic analysis showed that the African HEV strains were genetically divergent from those from Asia, and that all African strains were found on one branch when partial ORF1 was analysed. The endemic HEV strains identified in this study, both those causing sporadic infections in North Kordofan and those from the outbreaks in Darfur, were genetically similar to those causing outbreaks in Darfur in 2004 [26]. These strains formed a separate clade in the phylogenetic tree distinct from HEV strains from neighboring Chad, Egypt and also from strains from Namibia and Morocco. The North Kordofan and Darfur regions are large geographical regions, comparable to the area covered by Pakistan and Bangladesh together, or close to one tenth of India. Since the HEV strains isolated from Pakistan, Bangladesh and India are intermixed in the phylogenetic tree, and the Sudanese strains from all parts of this large region formed one clade, HEV strains circulating in India, Bangladesh and Pakistan may spread and evolve differently compared to those circulating in Sudan. This may partly be due to the higher population density of more susceptible individuals in the Asian countries, and that HEV strains may have circulated and evolved in these countries for a longer period than in Sudan. The interpretation of the phylogenetic analysis showing uniqueness of the HEV strains from Sudan may be limited because there are very few sequences available from HEV strains from different parts of Africa. Therefore, it is not known if the HEV strain identified in this study is specific to Sudan or may also be prevalent in other neighboring countries, and if there are several strains circulating in Sudan. However, the data obtained indicate that the endemic Sudanese strains have spread between different regions of the country for several years, probably mainly during rainy seasons. This endemic strain may be highly virulent, and therefore easily isolated, because of its ability to cause a large number of sporadic hepatitis cases also in younger age groups, and its high mortality. There is a need for complete genome sequencing of this strain to examine for the presence of known virulence markers [48,49] or other genetic markers that may explain the severity of hepatitis symptoms associated with this type. In addition, larger prospective studies on the HEV infection are needed during non-outbreak periods in all regions of Sudan, to identify whether this strain is prevalent in the whole country, whether it is associated with increased severity of hepatitis symptoms and whether it is causing outbreaks more often than other HEV1 strains.

## 5. Conclusions

Our study demonstrated that hepatitis E is a major public health issue in Sudan, even during non-outbreak periods. While resources for the control of communicable diseases are particularly difficult to use in these remote areas of Africa, there is a need for surveillance of hepatitis E for the early detection of upcoming and ongoing outbreaks. Efforts are needed to improve sanitation and access to clean water and safe latrines for most inhabitants in these remote regions. Cross-sectional serosurveys can also be implemented to estimate the susceptibility of the population to HEV and thus the risk of spread and future outbreaks. Multisectoral activities are needed in order to improve the access to clean drinking water and general sanitation for the population in these poor regions and for training in community hygiene and outbreak preparedness.

## Figures and Tables

**Table 1:** Origin and gender of patients with sporadic hepatitis infection in 12 different regions of the state of North Kordofan during 2011 and 2012 and of patients involved in hepatitis outbreaks in five regions of Darfur during the years 2012 and 2014.

Region		Number of Hepatitis E Virus (HEV)- Infected Individuals/Total Number of Individuals investigated		Total
		Men Anti-HEV IgM-positive/Total (%)	Women Anti-HEV IgM-positive/Total (%)	
**North Kordofan**	Alnohod	20/82 (24)	24/79 (30)	44/161 (27)
	Gibaish	1/25 (4)	3/18 (17)	4/43 (9)
	Wad Banda	6/41 (15)	10/54 (19)	16/105 (15)
	Abozabad	2/8 (25)	3/9 (33)	5/17 (29)
	Shaikan	0/23	2/16 (12)	2/39 (5)
	Al Khawai	2/5 (40)	15/19 (79)	17/24 (71)
	Sudari	0/2	0/3	0/5
	Bara	0/5	0/2	0/7
	West Bara	4/8 (50)	6/17 (35)	10/25 (40)
	Alrahad	0/1	0/1	0/2
	Omrawaba	0/1	0	0/1
	Alodeia	0/0	0/3	0/3
**Subtotal**		**35/211 (17)**	**63/221 (28)**	**98/432 (23)**
**Darfur**	North Darfur	21/30 (70)	1/4 (25)	22/34 (65)
	Central Darfur	4/29 (13)	0/6	4/35 (11)
	West Darfur	3/23 (13)	3/6 (50)	6/29 (21)
	East Darfur	0/0	1/6 (17)	1/6 (17)
	South Darfur	14/41 (34)	2/7 (28)	16/48 (33)
**Subtotal**		**42/123 (34)**	**7/29 (24)**	**49/152 (32)**
**TOTAL**		**77/334 (23%)**	**70/250 (28%)**	**147/584 (25%)**

**Table 2:** Clinical symptoms among 98 patients with sporadic hepatitis E virus infection.

Clinical Symptom	Number of Patients with Hepatitis E and Respective Symptom
Jaundice	97 (99%)
Fever	94 (96%)
Abdominal pain	72 (73.5%)
Loss of appetite	68 (69.4%)
Vomiting	72 (73.5%)
Joint pain	47 (48%)
Headache	32 (32.6%)

**Table 3:** Anti-HEV IgM and HEV RNA in relation to age of and number of investigated patients with clinical hepatitis in Kordofan and Darfur during the study period.

Region	Age (Years)	Men	Women	Total	HEV RNA Positive/Total Number Investigated
		Number of HEV-Infected Individuals/Total Number Investigated (%)	Number of HEV-Infected Individuals/Total Number Investigated (%)		
**North Kordofan**	<5	4/21 (19%)	1/11 (9%)	5/32 (16%)	4/5
	5–14	7/48 (15%)	18/48 (37%)	25/96 (26%)	9/25
	15–24	10/45 (22%)	20/55 (36%)	30/100 (30%)	9/30
	25–34	7/57 12%)	16/63 (25%)	23/120 (19%)	7/23
	35–44	3/15 (20%)	7/35 (20%)	10/50 (20%)	1/10
	45–54	3/13 (23%)	0/4	3/17 (17%)	1/3
	55–64	1/9 (11%)	0/2	1/11 (9%)	1/1
	>64	0/3	1/3 (33%)	1/6 (17%)	0/1
	**Total**	**35/211 (17%)**	**63/221 (28%)**	**98/432 (23%)**	**32/98**
**Darfur**	<5	0/5	0/4	0/9	0
	5–14	0/9	0/5	0/14	0
	15–24	16/47 (34%)	2/5 (40%)	18/52 (34%)	5/52
	25–34	21/50 (42%)	5/19 (50%)	26/60 (43%)	12/60
	35–44	4/7 (57%)	0/3	4/10 (40%)	1/10
	45–54	0/3	0/0	0/3	0
	55–64	1/1	0/0	1/1	0
	>64	0/1	0/2	0/3	0
	**Total**	**42/123 (34%)**	**7/29 (24%)**	**49/152 (32%)**	**18/152**
